# Breed-Dependent Transcriptional Regulation of 5′-Untranslated GR (NR3C1) Exon 1 mRNA Variants in the Liver of Newborn Piglets

**DOI:** 10.1371/journal.pone.0040432

**Published:** 2012-07-05

**Authors:** Huafeng Zou, Runsheng Li, Yimin Jia, Xiaojing Yang, Yingdong Ni, Rihua Cong, Paul D. Soloway, Ruqian Zhao

**Affiliations:** 1 Key Laboratory of Animal Physiology and Biochemistry, Ministry of Agriculture, Nanjing Agricultural University, Nanjing, People's Republic of China; 2 Division of Nutritional Sciences, Cornell University, Ithaca, New York, United States of America; Kyushu Institute of Technology, Japan

## Abstract

Glucocorticoids are vital for life and regulate an array of physiological functions by binding to the ubiquitously expressed glucocorticoid receptor (GR, also known as NR3C1). Previous studies demonstrate striking breed differences in plasma cortisol levels in pigs. However, investigation into the breed-dependent GR transcriptional regulation is hampered by lacking porcine GR promoter information. In this study, we sequenced 5.3 kb upstream of the translation start codon of the porcine GR gene, and identified seven alternative 5′-untranslated exons 1–4, 1–5, 1–6, 1–7, 1–8, 1–9,10 and 1–11. Among all these mRNA variants, exons 1–4 and 1–5, as well as the total GR were expressed significantly (*P*<0.05) higher in the liver of newborn piglets of Large White (LW) compared with Erhualian, a Chinese indigenous breed. Overall level of CpG methylation in the region flanking exons 1–4 and 1–5 did not show breed difference. However, nuclear content of Sp1, p-CREB and GR in the liver was significantly (*P*<0.05) higher in LW piglets, associated with enhanced binding of p-CREB, and higher level of histone H3 acetylation in 1–4 and 1–5 promoters. In contrast, GR binding to promoters of exons 1–4 and 1–5 was significantly diminished in LW piglets, implicating the presence of negative GREs. These results indicate that the difference in the hepatic expression of GR transcript variants between two breeds of pigs is determined, at least partly, by the disparity in the binding of transcription factors and the enrichment of histone H3 acetylation to the promoters.

## Introduction

Glucocorticoids (GCs) are involved in the regulation of almost all the biological processes ranging from growth and development to immunity and reproduction [1], [2]. The biological functions of GCs require the functionality of their intracellular binding protein, glucocorticoid receptor (GR) which is also known as nuclear receptor subfamily 3, group C, member 1 (NR3C1). GR is expressed in almost all the cells studied. The intracellular concentration of GR protein varies greatly among cell types and determines, in large part, the cellular responses to the hormone. Therefore, processes that regulate the expression of GR gene are critical to the maintenance of appropriate functions of glucocorticoids.

The GR gene spans more than 80 kb and is conserved among humans, rats and mice [3], [4]. Human GR contains nine untranslated alternative first exons and eight coding exons (exons 2–9) [3]. Since the alternative first exons are each preceded by their own promoter, the tissue-specific usage of these alternative promoters results in different GR mRNA transcripts [4]. The 5′-heterogeneity of GR mRNA transcripts and promoter usage represent complex mechanisms for the regulation of GR transcription in different tissues under different conditions. Among the nine untranslated alternative first exons, seven are located in the proximal promoter region approximately 5 kb upstream of exon 2. The proximal GR promoter comprises of a CpG island which shows high sequence homology between rats and humans [5].

A wide variety of transcription factors have been identified to bind to their cis-acting elements or binding sites on the CpG island of GR promoter to regulate GR transcription, among which are specificity protein 1 (Sp1) [6], YinYang 1 (YY1) [7], cAMP response element binding protein (CREB) [8], Nerve growth factor-inducible protein A (NGFI-A) [9] and activator protien-1 (AP-1) [10]. GR, a transcription factor itself, works together with other transcriptional factors to fine-tune its own transcription. The auto-regulation of GR transcription can be stimulatory or inhibitory, depending on the nature of the glucocorticoid response elements (GREs). Several positive GREs or negative GREs (nGREs) have been identified in CpG island promoters of human, rats or mice GR genes [11], [12].

Epigenetic mechanisms, such as DNA methylation and histone modification, act in concert with transcription factors to regulate GR transcription. For instance, low maternal care increases the CpG methylation of the NGFI-A binding site in the GR promoter 1–7, which is associated with the inhibition of histone acetylation and NGFI-A binding to GR 1–7 promoter in the hippocampus of offspring rats. These epigenetic alterations subsequently cause lower GR expression in the hippocampus and higher stress responses in the offspring of low caring mothers [13]. Epigenetic programming of GR is also reported in peripheral tissues. Maternal dietary protein restriction during pregnancy leads to decreased methylation of GR 1–10 promoter and increased GR expression in the liver of rat offspring [14].

Our knowledge on GR gene and its transcriptional regulation is mostly based on studies in humans, mice and rats [3]. Pigs serve as excellent model for human metabolic research because they share high similarity to humans in anatomy, physiology, development, metabolism, and omnivorous habits [15]. Previous studies demonstrate striking breed differences in plasma cortisol concentration and GR mRNA expression in pigs [16], [17], Chinese indigenous Erhualian (EHL) pigs demonstrate 2-fold higher plasma cortisol concentration than Pietrain pigs [18]. Moreover, GR expression in hippocampus [17], liver and muscle [16] also differs between Chinese and Western pig breeds, which is associated with the breed-specific characteristics in stress responses, hepatic gluconeogenesis, and intramuscular fat deposition, respectively. Accumulating evidence suggests that dysfunction of hepatic GR is involved in the development of metabolic diseases including obesity, diabetes and fatty liver [19], [20]. Studies on the regulation of hepatic GR expression in the pig may shed light on the pathogenesis of GR related metabolic disorders in human liver. However, investigation into GR transcriptional regulation in the pig is hampered by lacking genomic information on porcine GR promoters.

Therefore, the present study was aimed first, to clone and sequence the porcine GR promoters; second, to compare the hepatic expression of 5′-untranslated GR first exon mRNA variants between two pig breeds differing in plasma cortisol concentrations and; third, to investigate the breed-dependant transcriptional regulation of GR in porcine liver.

## Results

### Porcine GR gene promoter demonstrated higher homology to human than the rat

**Figure 1:**
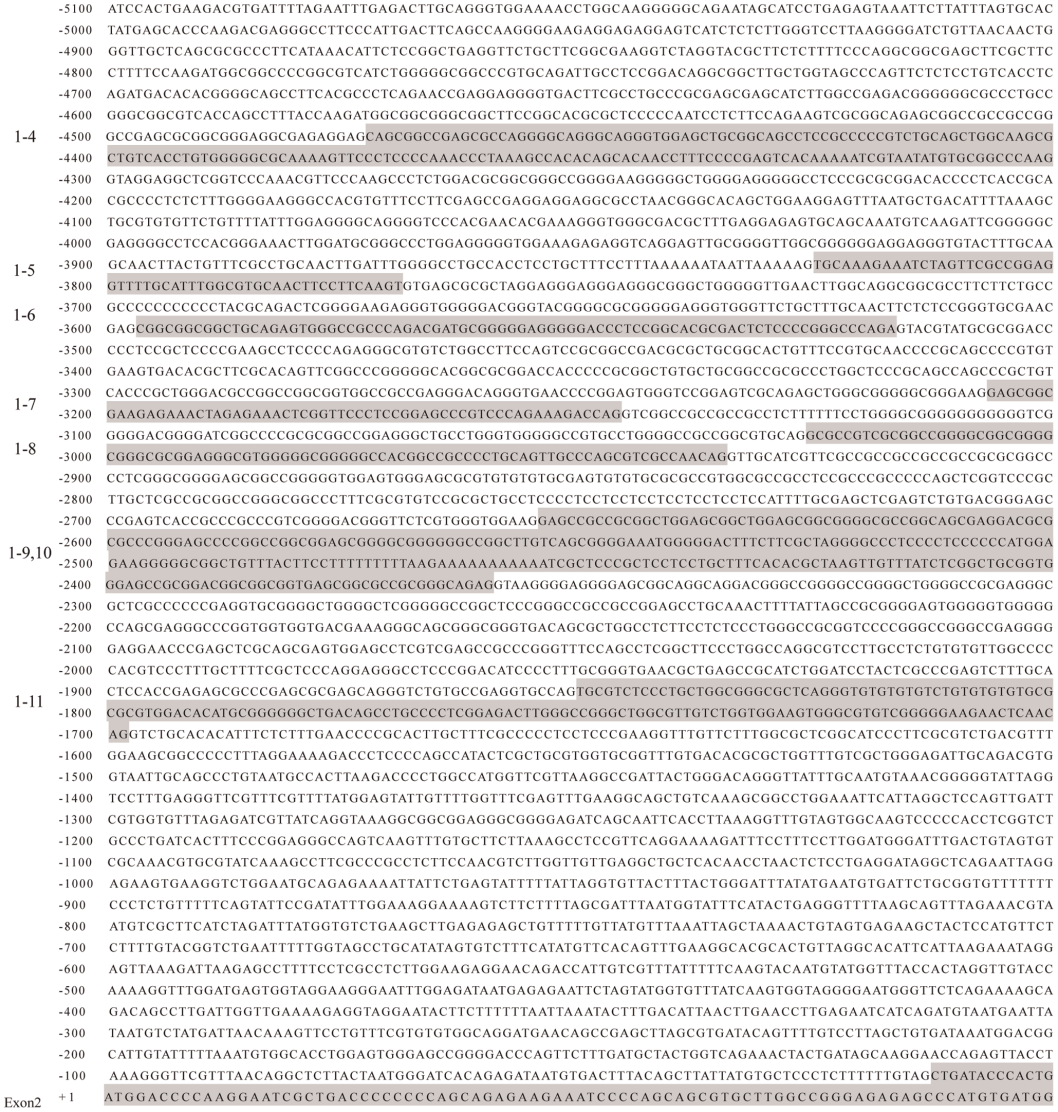
DNA sequence of the pig GR gene flanking the 5′-end of exon 2. Numbering is with respect to the translation start site within exon 2. The start of the porcine exon 2 is at −13 bp. Shaded sequences represent identified porcine alternative GR first exons.

We first compared the published human (AY436590), rat (AJ271870) and mouse (X66367) GR promoter sequences and identified the highly conserved regions. Using these highly conserved sequences and the porcine GR cDNA sequence (AY779185.1) as probes, we screened the porcine BAC library and hit a positive clone (CH242–105G5). The BAC clone was then priority sequenced by the Sanger Institute upon our request. Within the complete sequence (CU928713) of the clone, we verified, by sequencing the overlapping PCR products amplified from porcine genomic DNA extracted from liver, a 5300 bp 5′ flanking sequence of porcine GR exon 2 (Fig. 1). Using primers shown in Table 1, we identified seven untranslated alternative exons (1–4, 1–5, 1–6, 1–7, 1–8, 1–9,10, and 1–11) with RT-PCR in porcine total RNA extracted from various types of tissues.

**Table 1:** Body weight, liver weight and serum cortisol concentrations in newborn piglets.

Parameters	LW	EHL	*P* value
Body weight (kg)	1.31±0.04	0.75±0.03	<0.01
Liver weight (g)	31.76±1.72	17.16±1.21	<0.01
Relative liver weight (%)	24.27±0.91	23.04±2.01	>0.05
Serum cortisol (ng/ml)	154.11±22.57	315.45±41.54	<0.01

Values are mean ± SEM, n = 6.

**Figure 2:**
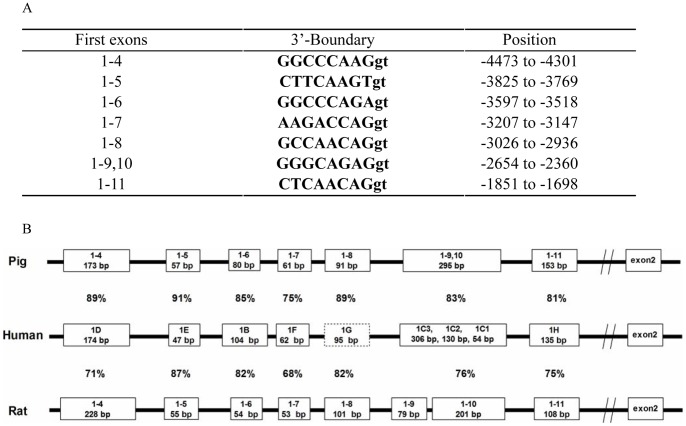
Characteristics of first exons of porcine GR. (A) Locations of the 3′ boundary of the porcine alternative first exons of porcine GR. (B) Map of the alternative first exons of the porcine GR gene and their human and rat homologues. Experimentally identified exons are shown in solid line boxes and the predicted human GR exon 1-G is shown in dotted box. The size of the exons shown is not to scale. Length of each exon is shown in the respective box in base pairs, and the numbers in between represent the percentage homology of respective exons in comparison with the human homologue.

The 3′ boundaries of the seven alternative first exons were determined (Fig. 2A) and the sequences were aligned with the relevant regions of rat (AJ271870) [21], and human (AY436590) [4] for homology analysis. Compared to the rat sequence, the alternative first exons of the porcine GR showed greater homology with that of the human GR gene (Fig. 2B). Moreover, similar to human GR gene, the exon 1–9,10 of porcine GR is a single exon, while in rats exon 1–9 and exon 1–10 are 2 independent exons separated by an intron [3].

The reported nucleotide sequence of the porcine GR promoter is published in NCBI database with the accession number JN624280.

### Body weight, liver index and serum cortisol concentration were significantly different between Large White (LW) and Erhualian (EHL) piglets at birth

As shown in Table 1, LW piglets were about two-fold heavier in body weight than EHL piglets at birth (*P*<0.01). Accordingly, liver weight was also significantly higher in LW piglets (*P*<0.01), yet the liver index (liver weight relative to body weight) did not differ between breeds. Serum cortisol concentration was two-fold higher in EHL piglets compared with LW piglets at birth (*P*<0.01). These findings are in agreement with the previously published results of breed comparison studies in pigs [18], [22].

### Hepatic expression of total GR mRNA and 5′-untranslated GR Exon 1 mRNA variants were different between LW and EHL piglets at birth

We determined the abundance of total GR mRNA and the first alternative exon mRNA variants in the liver of newborn piglets with qRT-PCR using primers shown in Table 2. All the seven GR first exon transcript variants were expressed in the liver of newborn piglets. The abundance of GR exon 1 mRNA variants was estimated relative to that of the total GR mRNA, using 18S as an internal standard.

**Table 2:** Nucleotide sequences of specific primers used in the study.

GR Alternative first Exons	Primers (from 5′ to 3′)	Product (bp)	Application
Exon 1–4	F: CACACAGCACAACCTTTC	161	qRT-PCR for quantitation of GR exon 1 mRNA variants
	R: AACCTTCACAGGAGTTCC		
Exon 1–5	F: GCGTGCAACTTCCTTCAA	207	
	R: CTTGGAGTCTGGCTGAGA		
Exon 1–6	F: GAGTGGGCCGCCCAGACGAT	189	
	R: CCTCCCCTCAGGCTTTTAT		
Exon 1–7	F: GCGAAGAGAAACTAGAGAAA	185	
	R: AACCTTCACAGGAGTTCC		
Exon 1–8	F: TGCCCAGCGTCGCCAACA	144	
	R: CCTCCCCTCAGGCTTTTAT		
Exon 1–9,10	F: CCTGCTTTCACACGCTAA	177	
	R: ATCACATGGGCTCTCTCC		
Exon 1–11	F: CTGGTGGAAGTGGGCGTGTC	163	
	R: TTCCTCCCCTCAGGCTTTTAT		
Total GR	F: CCAAACTCTGCCTTGTGTGTTC	108	
	R: TGTGCTGTCCTTCCACTGCT		
18S	F: CCCACGGAATCGAGAAAGAG	122	
	R: TTGACGGAAGGGCACCA		
Promote 1–4	F: AGAACCGAGGAGGGGTGACT	142	ChIP for detecting transcription factor binding
	R: CTGGAAGAGGATTGGGGGAG		
Promote 1–5	F: CTGCGTGTGTTCTGTTTTAT	166	
	R: CAACTCCTGACCTCTCTTTC		

**Figure 3:**
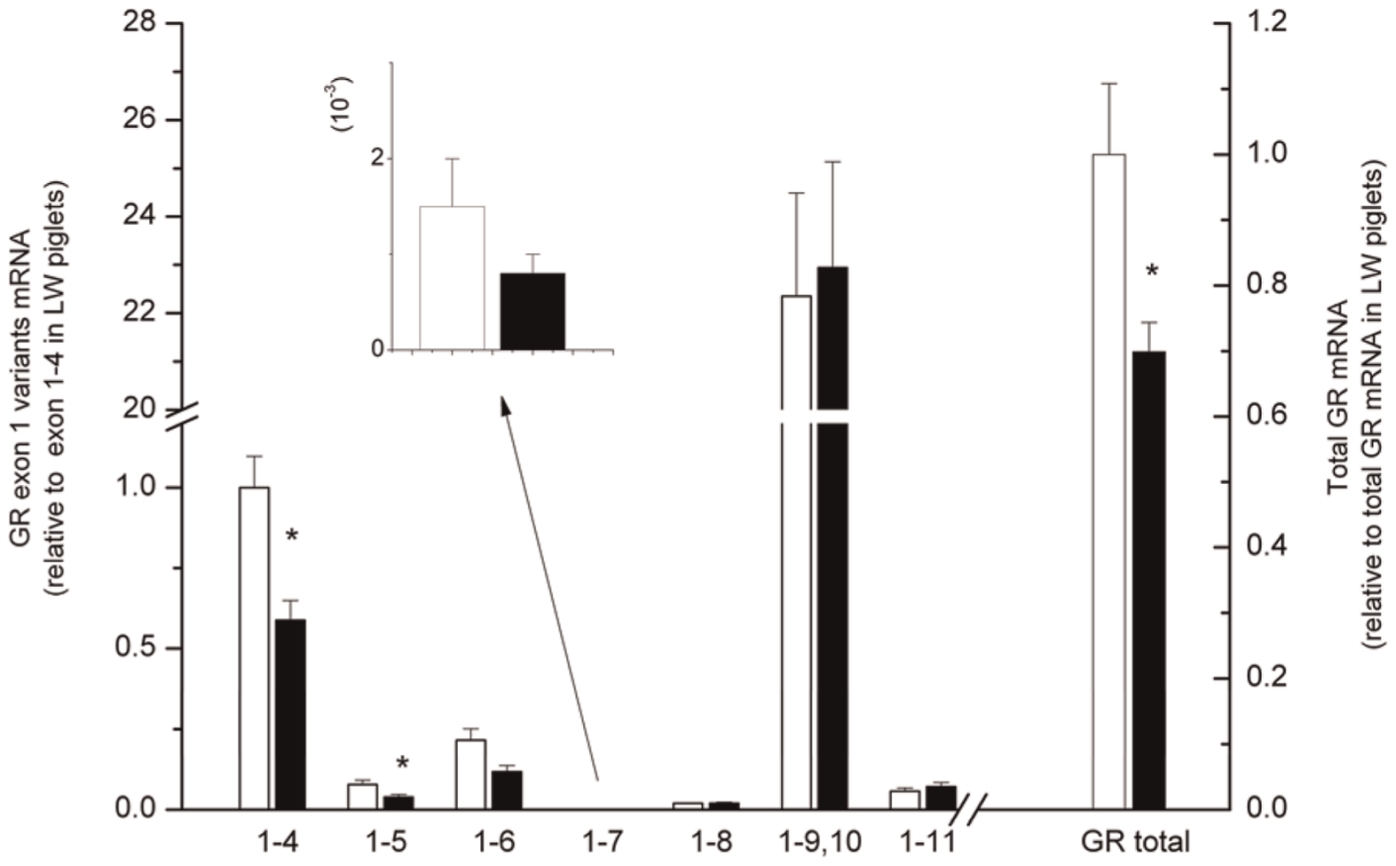
Hepatic expressions of total GR mRNA and 5′-untranslated GR exon 1 mRNA variants. Blank bars represent Large White (LW) piglets; filled bars represent Erhualian (EHL) piglets. Results shown are the fold changes relative to the average value of the GR exon 1–4 mRNA in the liver of LW piglets. Data are presented as mean ± SEM. * indicates significant difference between breeds (*P*<0.05).

Among seven GR first exon variants, the most abundant variant expressed in the liver was exon 1–9,10, which was followed by exons 1–4, 1–6, 1–5, 1–11, 1–8 and 1–7 in descending order. GR exons 1–4 (*P*<0.01) and 1–5 (*P*<0.05), as well as the total GR (*P*<0.05) mRNAs were expressed in a breed-dependent manner in the liver, newborn LW piglets showing significantly higher expression compared with EHL piglets (Fig. 3).

### Methylation levels of some CpG sites on porcine GR promoter differ between breeds

**Figure 4:**
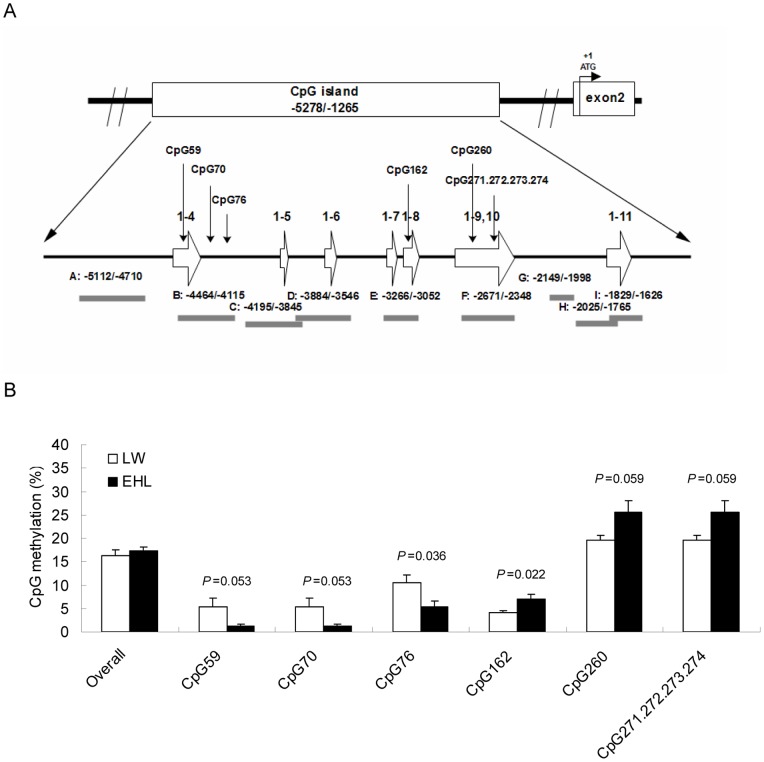
Methylation status of the proximal GR promoter of pig GR gene. (A) Location of the CpG island, the alternative first exon, and the differently methylated CpG sites on the proximal region of porcine GR promoter. Bars underneath represent the location of the 9 pairs of primers (A to I) used for Sequenom's Mass ARRAY analysis. The locations of the amplicons are also numbered with respect to the translation start site within exon 2. (B) Quantitation of the overall methylation levels across the CpG island of the proximal GR promoter and the methylation levels of individual CpG sites showing breed difference. Data are presented as mean ± SEM. P values are shown above the bars demonstrating the significance of the breed differences.

Using Methyl Primer Express@ V1.0 (ABI, USA), we predicted a CpG island in the porcine GR gene promoter at positions −5278 to −1265 upstream of the ATG translation start codon in exon 2 (Fig. 4A). All the seven alternative first exons are located in this CpG island. We analyzed the level of methylation on each CpG site located within the 4014 bp CpG island of porcine GR promoter using SEQUENOM's MassARRAY method. Nine pairs of primers covering almost the entire length of the CpG island were used. The raw data from MassARRAY analysis indicating the methylation rates of each CpG units are shown as Table S1. The average percentages of CpG methylation across the whole length of GR promoter were similar in the liver of LW and EHL piglets at birth (Fig. 4B). Neither the intronic nor the exonic regions of each alternative first exons showed significant breed differences in the level of CpG methylation (Data not shown). However, breed differences occurred on some specific CpG sites (Fig. 4B). The methylation rates on CpG 59 (*P* = 0.05), 70 (*P* = 0.05), and 76 (*P*<0.05) were higher in LW than in EHL pigs, whereas the opposite was true for CpG 162 (*P*<0.05), 260 (*P* = 0.06), and CpGs 271∼274 (*P* = 0.06). As CpG sites 271∼274 are located in close proximity, the MassARRAY method could only report the methylation rate collectively for these 4 adjacent sites. CpG sites 59, 70 and 76 are located in the promoter of exon 1–5, whereas CpGs 260 and 271∼274 are located in the promoter of exon 1–11. The breed disparities on the methylation rates of these CpG sites were not in accordance with the differential expression of the corresponding GR mRNA variants in the liver of the two pig breeds [18], [22].

### Hepatic expression of Sp1, p-CREB and GR in newborn piglets was breed-specific

**Figure 5:**
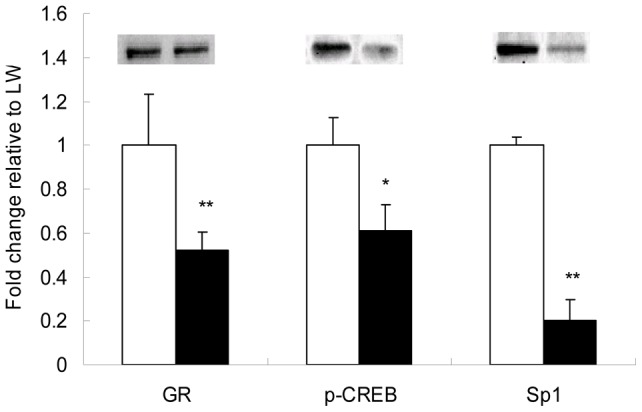
Western blot analysis of GR, p-CREB and Sp1 in liver nuclear protein extracts of LW and EHL pigets. Histone H1 was used as a loading control. The nuclear contents of the transcription factors are presented as the fold change relative to LW. Blank bars represent Large White (LW) piglets; filled bars represent Erhualian (EHL) piglets. Values are presented as mean ± SEM. ** donates *P*<0.01 and * donates *P*<0.05, compared with LW.

The cis-acting elements of transcription factors Sp1 [6], CREB (7) and GR [12] are located in human GR promoter 1–4 and 1–5 and involved in the transcriptional regulation of GR gene. To clarify whether these transcriptional factors are expressed in a breed-dependant manner, we analyzed the nuclear abundance of these transcriptional factors in the liver of newborn LW and EHL piglets. Western blot analysis revealed that the nuclear abundance of Sp1, p-CREB and GR in the liver was significantly (P<0.05) higher in LW piglets (Fig. 5).

### Binding of p-CREB and GR to GR exon 1–4 and 1–5 promoters was breed-specific, which was associated with breed difference in histone H3 acetylation (H3Ac)

**Figure 6:**
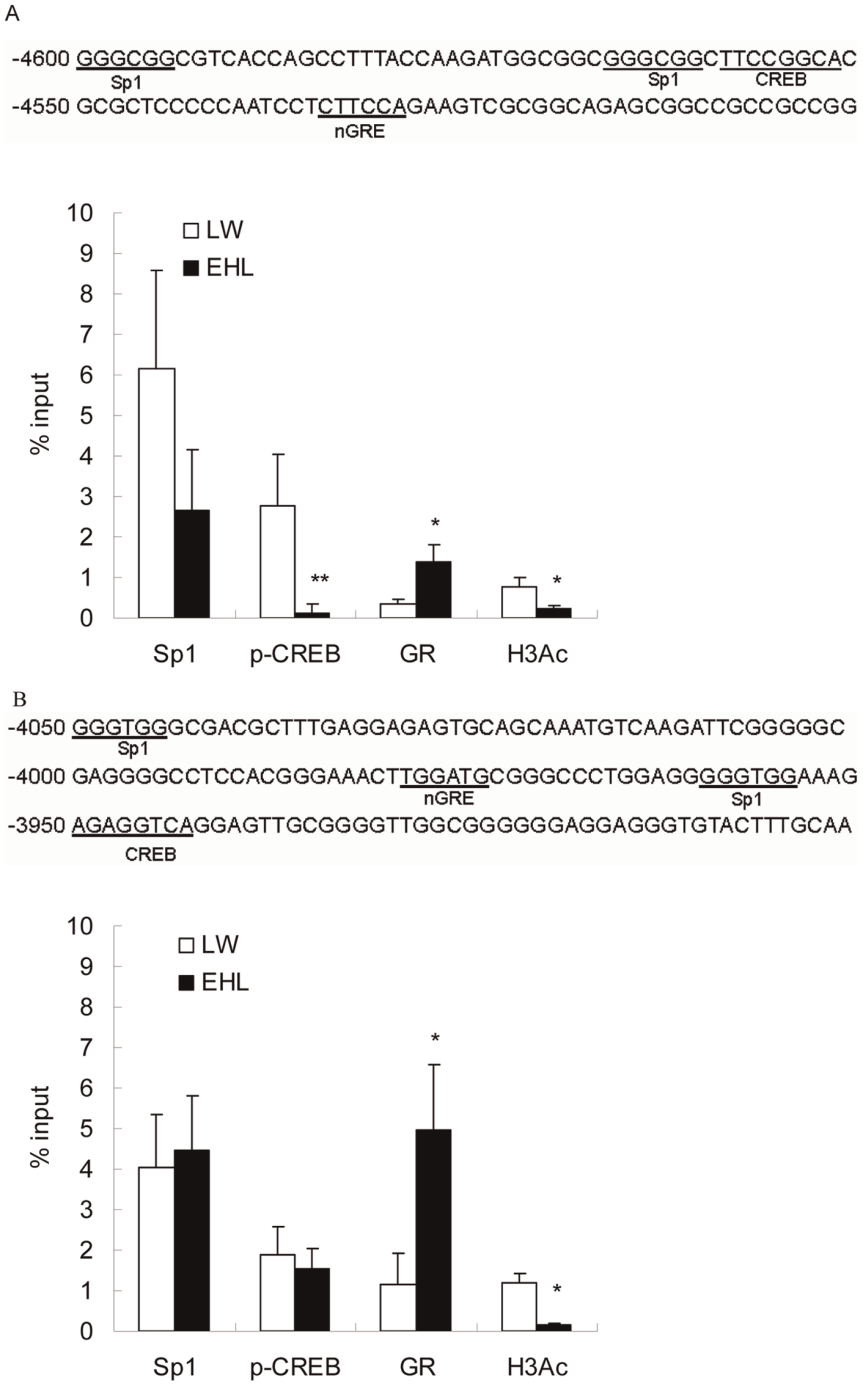
ChIP analysis of transcription factors binding to GR promoter 1–4 (A) and 1–5 (B). Nucleotide sequences of the PCR products for GR 1–4 and 1–5 promoters are shown above the respective bar graph. Predicted binding sites of Sp1, CREB and GR are underlined. Data are shown as a percent of the input DNA which represents the amount of chromatin used in the ChIP analysis. Data are presented as mean ± SEM. ** donates *P*<0.01 and * donates *P*<0.05, compared with LW.

We identified one CREB and one Sp1 binding motif in promoter 1–4, and one CREB, two Sp1 binding sites in promoter 1–5. 5′-GGTACAnnnTGTTCT-3′ has been recognized as a positively modulated glucocorticoid responsive element (GRE) [23], whereas the consensus sequence 5′-tggacg-3′ was suggested as negatively modulated glucocorticoid responsive element (nGRE) [12], [24]. No GRE was found, however two sequences, 5′-cttcca-3′ and 5′-tggatg-3′, with only one nucleotide substitution compared to the complementary sequence of nGRE and nGRE, respectively, were identified in porcine alternative GR 1–4 and 1–5 promoters (Fig. 6).

To clarify whether the predicted cis-acting elements on porcine GR 1–4 and 1–5 promoters are involved in the regulation of the breed-dependent expression of GR exon 1–4 and 1–5 variants, chromatin immunoprecipitation (ChIP) analysis was used to determine the binding of p-CREB, Sp1 and GR to the corresponding regions of the promoters. LW piglets demonstrated significantly (*P*<0.01) higher p-CREB binding to GR promoter 1–4 (Fig. 6A), yet GR bindings to both promoter 1–4 and 1–5 were significantly (*P*<0.05) diminished in the liver of LW piglets (Fig. 6). The higher p-CREB binding to GR promoter 1–4 in LW piglets correlated with higher expression of GR exon 1–4 in this breed, implicating a positive role of p-CREB in the regulation of GR exon 1–4 transcription. On the contrary, LW piglets expressing higher GR exon 1–4 and 1–5 mRNA variants exhibited diminished GR binding to the respective promoters, supporting the prediction of negative GREs in porcine GR 1–4 and 1–5 promoters.

Moreover, ChIP assay revealed significantly higher (*P*<0.05) level of histone H3 acetylation in promoter 1–4 (*P*<0.05) and 1–5 (**P**<0.01) in LW piglets, which is in line with the higher abundances of GR exon 1–4 and 1–5 mRNA variants detected in the liver of LW piglets (Fig. 6). This finding agrees with the common notion that histone acetylation is usually associated with transcriptional activation [25].

## Discussion

To our knowledge, this is the first report on the cloning and analysis of the proximal promoter sequence of porcine GR gene. Our results agree with the previous notion that GR promoter is highly conserved among mammalian species studied, including human [4], rat [21], mice [26], etc. It is worth noting that pig is more close to human than the rodents in terms of the sequence homology and structure similarity of the GR promoter, implying that pig can serve as a better model, compared to the rodents, for human metabolic diseases related to the dysregulation of GR expression. Although the nomenclature used to name the alternative first exons differs between human and pig, the seven alternative first exons identified in pig GR promoter match well with the corresponding counterparts reported in human GR promoter [27]. Nevertheless, we noticed minor differences in human GR exon 1G and 1C, in comparison with porcine exon 1–8 and 1–10 respectively. Human exon 1G was predicted from the sequence homology analysis with the rat exon 1–8 [4], but has not been experimentally validated. The pig GR exon 1–8 identified in this study demonstrated high similarity (89% homology) with the predicted human GR exon 1G, further supporting the existence of exon 1G in human GR gene. Human GR exon 1C is reported to have three variants, 1C1, 1C2 and 1C3 [28], but we failed to identify similar variants for the corresponding exon 1–9,10 in pig GR promoter. No information is available on the functional significance of the exon 1C variants in human GR promoter.

Numerous studies have demonstrated disparity in GR expression between different breeds or strains of rats [29], mice [30], chickens [31], [32] and pigs [16], [17], [33], [34]. Majority of the studies focused on GR expression in the brain, while some concern GR expression in peripheral tissues such as liver [31], muscle [16], adipose tissue [33] and placenta [34]. Here we found that total GR mRNA expression was significantly lower in the liver of newborn EHL piglets, which was associated with higher serum cortisol level. Such negative correlation between hepatic GR expression and blood corticosterone level agrees with the findings in Italian chicken breeds [31], yet contradicts to the previous reports on porcine muscle [18] and adipose tissue [33], where Chinese indigenous pigs, EHL or Meishan, demonstrated higher GR expression at later ages. Therefore, the breed-specific GR expression in the pig appears to be dependent on the age and/or tissue-type.

Studies on human and rodents indicated that the 5′-heterogeneity of GR mRNA transcripts are tissue-specific and regulated by complex mechanisms under different conditions [21], [28]. We report here, for the first time, the breed-specific expression of GR exon 1 mRNA variants in porcine liver. Among seven untranslated GR first exon transcripts, only exon 1–4 and 1–5 mRNA variants expressed differently between breeds, in a same fashion as total GR mRNA. A similar phenomenon was observed in rat hippocampus concerning GR exon 1–7. Maternal care resulted in up-regulation of hippocampal expression of GR exon 1–7 transcript, which was accompanied by increases of total GR mRNA and protein [13]. In another study, adult rats treated with antidepressant drug Fluoxetine expressed higher GR exon 1–7 together with total GR mRNA in hippocampus, while the constitutional GR mRNA variant, exon 1–10, remained unchanged [35]. However, exon 1–4 and 1–5 mRNA variants, which expressed in much lower abundances compared to the constitutive exon 1–9/10, are unlikely to be the main and only contributors for the breed differences in total GR expression. It is noted that, apart from the proximal exon 1 mRNA variants detected in this study, there might be also distal exon 1 variants and/or unidentified variants expressed in the liver. The percentages of these variants are not determined and it is unknown whether some of them are expressed in a breed dependant manner.

Previous studies suggest that the methylation status of some specific CpG sites in the CpG island of GR promoter can significantly affect transcription of GR mRNA variants, such as exon 1–7 transcript in hippocampus [36], and exon 1–10 transcript in liver [14]. Maternal protein restriction during pregnancy changed the level of methylation in PPAR-α gene promoter in the liver of juvenile rat offspring [37]. Based on the consideration that EHL pigs were traditionally raised under low protein diet, we speculated that the methylation level of GR promoter of EHL may differ from LW piglets. However, we failed to detect breed differences in the overall level of CpG methylation for either the whole length of GR promoter or the proximal promoters for each of alternative first exons. It is possible that methylation changes on some CpG sites in GR promoter would be adequate to fine-tune GR transcription. Indeed, some CpG sites were found to be differently methylated between breeds, among with are CpG70 and 76 containing predicted binding sites for transcription factors, Sp1 and GR.

Transcriptional control within the CpG island of human GR promoter has been well characterized [11]. Here we provide the first evidence that Sp1, CREB and GR could translocate to the nucleus and bind to porcine GR exon 1–4 and 1–5 promoters. Moreover, we found breed disparities not only in nuclear content of GR, Sp1 and CREB, but also in the binding of CREB to GR exon 1–4 promoter and GR to both exon 1–4 and 1–5 promoters. Sp1 and CREB are reported to positively regulate gene transcription, while GR can regulate gene transcription either positively or negatively [38]. Enhanced binding of p-CREB to promoter 1–4 in LW piglets was associated with higher hepatic GR exon 1–4 transcript, which is in agreement with the previous reports [12]. However, binding of GR to promoter 1–4 and 1–5 was negatively correlated with the abundance of GR exon 1–4 and 1–5 transcripts, which supports the prediction of nGREs in porcine GR promoters. This negative feedback regulation of GR on the transcription of itself may explain, to some extent, why high serum cortisol level in EHL piglets was associated with lower hepatic GR expression.

Histone acetylation causes relaxation of chromatin structure and increases the recruitment of transcription factors and RNA polymerase II to initiate transcription [39], thus is often associated with gene activation. Indeed, higher expression of GR exon 1–4 and 1–5 mRNA variants detected in LW piglets was accompanied by increased enrichment of acetylated histone H3 on their promoters. Recent epigenetic studies suggest that DNA methylation, histone modifications, and the binding of transcription factors on a specific locus are closely related and work in concert to regulate gene transcription [40]. Further analytical studies are required to decipher the interactions among these regulatory elements and to evaluate their contributions to the breed-specific expression of 5′-untranslated GR first Exon mRNA variants in the liver of newborn piglets.

Taken together, our results indicate that the difference in the hepatic expression of GR exon 1–4 and 1–5 transcript variants between two breeds of pigs is determined, at least partly, by the disparity in the binding of CREB and GR, as well as the enrichment of histone H3 acetylation to the GR exon 1–4 and 1–5 promoters. Methylation of some CpG sites may also contribute to the transcriptional regulation of GR in the liver of newborn piglets. The genomic information on the promoter of porcine GR gene may provide essential basis for further investigations into GR gene regulation in the pig for both agricultural and human medical research.

## Materials and Methods

### Animals

To minimize the interference of postnatal factors such as nutrition (milk yield and milk composition) and maternal behavior, we chose to use newborn piglets in the present study. The newborn piglets were obtained from two neighboring pig breeding farms in Changzhou, Jiangsu Province, China. Six newborn male piglets from three litters (2 from each litter) of purebred EHL and LW sows, respectively, were sacrificed within 5 minutes after birth by exsanguinations. Body weight and liver weight were recorded. The blood was collected from the precaval vein and the serum was gathered and kept at −20°C. Liver samples were harvested within 10 min after slaughter and immediately frozen in liquid nitrogen, and then kept at −80°C until subsequent analysis. The slaughter and sampling procedures complied with the “Guidelines on Ethical Treatment of Experimental Animals” (2006) No. 398 set by the Ministry of Science and Technology, China and the “Regulation regarding the Management and Treatment of Experimental Animals” (2008) No.45 set by the Jiangsu Provincial People's Government. The experimental protocol was specifically approved by the Animal Ethics Committee of Nanjing Agricultural University.

### Radioimmunoassay for serum hormone levels

Cortisol was measured with commercial RIA kits purchased from Beijing North Institute of Biotechnology, Beijing, China. The detection limit for cortisol was 2 ng/mL and the intra- and inter-assay coefficients of variation were 10% and 15%, respectively. Samples were analyzed in duplicate within one assay to avoid inter-assay variations.

### RNA extraction and GR mRNA exon 1 variant quantification

Total RNA was extracted from liver using TRNzol Total RNA Kit (Tiangen Biotech Co. Ltd, Beijing, China), according to the recommendations of the manufacturer. Two micrograms of total RNA were reverse-transcribed with M-MLV reverse transcriptase (Promega, Madison, WI, USA) following the standard protocol. Using transcript-specific primer pairs (Table 1), we measured the expression of these alternatively spliced transcripts and the total GR mRNA in liver of LW and EHL piglets. Real-time PCR was performed on a Mx3000P (Stratagene, La Jolla, CA, USA) with SYBR Premix Ex Taq™ (TaKaRa, Dalian, China) to quantitate mRNA of total GR and its 5′ first exon variants. The primers were designed to ensure that same PCR condition (denaturing at 95°C for 2 min, followed by 40 cycles of 95°C for 20 s, 64°C for 20 s, 72°C for 20 s, and then 72°C for 10 min) can be used to amplify all the GR mRNA variants with a similar efficiency. To confirm the amplification specificity, the PCR products from each primer pair were subjected to a melting curve analysis and sequencing.

### Identification of 5′ and 3′ boundary locations of the alternative first exons of pig GR gene

The 3′ boundaries of the alternative first exons were determined by RT-PCR followed by sequencing. The upstream primers, which were designed according to the sequence homology of pig with human and rat, were located in various first exons, while the downstream primers were located in coding region of exon 2. Seven pairs of primers were used to amplify the alternative first exons with cDNAs, then the PCR products were detected in gel and the expected bands were cut out and cloned into the pMD®18-T Vector (TaKaRa, Dalian, China), after that the clones were sequenced. All the alternative first exons were spliced to the same nocleotide sequence located in exon 2, thus the 3′ end of the first exons were determined.

The 5′ boundaries were identified by bioinformatic prediction referring to the 5′ boundary sequences of the first exons in human and rat GR. All the spliced introns obeyed the GT/AG rule.

### Sequenom's Mass ARRAY for quantitative methylation analysis of porcine GR promoter

Genomic DNA was treated with sodium bisulfite using EZ-96 DNA methylation kit (Zymo Research). Sequenom's Mass ARRAY platform (CapitalBio, Beijing, China) was used to perform quantitative methylation analysis. The system utilizes MALDI-TOF mass spectrometry in combination with RNA base-specific cleavage (MassCLEAVE). A detectable pattern is then analyzed for methylation status. PCR primers were designed by using Methprimer (www.urogene.org/methprimer/). For each reverse primer, an additional T7 promoter tag for in vitro transcription was added. In addition, a 10-mer tag was added on each forward primer to adjust for melting-temperature differences between the forward and reverse primers. The sequences of the primers used are not shown here and will be available upon request.

Computer program based analysis of GR promoter 1–4 and 1–5.

Two internet based programs, namely Transcription Element Search Site TESS (http://www.cbil.upenn.edu/tess) and TFSEARCH (http://www.cbrc.jp/research) were used to identify putative cis-element binding sites present in GR exon 1–4 and 1–5 promoters. Nucleotide sequences having higher matrix scores (80%) were considered as probable cis-acting elements [41].

### ChIP assay for the analysis of transcription factors binding and histone 3 acetylation on GR promoters

Antibodies against GR (sc-1004, Santa Cruz, CA, USA), Sp1 (sc-14027, Santa Cruz, CA, USA), p-CREB (BS4053, Bioworld Technology, MN, USA), and acetyl-Histone H3 (06-599, Millipore, MA, USA) were used for chromatin immunoprecipitation assay following previously described procedure [42]. Briefly, approximately 300 mg frozen liver tissue was ground in liquid nitrogen and fixed by addition of formaldehyde to a final concentration of 1% for 10 min at room temperature. The reaction was stopped by adding glycine to a final concentration of 125 mM while keeping rotating for 5 min at room temperature. The pellet was resuspended in cell lysis buffer (10 mM Hepes, pH 7.9, 1.5 mM MgCl2, 10 mM KCl, 0.2% NP-40, 0.2 mM sodium orthovanadate, 0.15 mM spermine, 0.5 mM spermidine, 1 mM EDTA, 5% sucrose, 1 mM dithiothreitol, and protease inhibitors), Dounce homogenized, and centrifuged. The pellet was washed once with ice cold PBS and resuspended in 3 ml SDS lysis buffer (50 mM Tris-HCl, 10 mM EDTA, 1% SDS). The crude chromatin released from the nuclei was sonicated (Sonic & Materials Inc., USA) at 50% amplitude for 3 min (10 s on, 10 s off) to produce 500-1000 bp fragments. Sonicated chromatin was quantified on the basis of DNA content at A260. The chromatin equivalent of 40 µg DNA based on the absorption at A260 was used in each immunoprecipitation. One-fifth of chromatin used for each immunoprecipitation was saved and designated as input. Three micrograms of respective antibody or normal IgG (control) were added to the chromatin and incubated overnight at 4°C. The immune complexes were collected using protein A/G plus beads (SC-2003, Santa Cruz, CA, USA), followed by extensive washing with low salt, high salt and LiCl buffers, and then twice with TE buffer (pH 8.0). Then the DNA-protein-antibody complexes were eluted and the DNA-protein formaldehyde cross-links were reversed by incubation at 65°C for 6 h. Proteins were digested by proteinase K at 45°C for 2 h. DNA fragments were purified by phenol/chloroform extraction and ethanol precipitation in the presence of 2 µl 10 mg/ml glycogen. DNA pellet was resuspended in 30 µl of H2O. Three micro liters of ChIPed DNA were used as the PCR template. The primers used for PCR amplification were shown in Table 2.

The amount of precipitated DNA was calculated relative to the input, The results are represented as a percentage of the input DNA (% input), which is calculated as 2 ^−△Ct [normalized ChIP]^×100, where △Ct_ [normalized ChIP]_  =  Ct _[ChIP]_ – (Ct _[Input]_ – Log_2_ (Input Dilution Factor)) [43].

### Western blot analysis for measuring the nuclear content of transcription factors

Nuclear protein extracts was prepared from liver samples as previously described [44]. Briefly, about 200 mg frozen liver tissue was ground in liquid nitrogen and rresuspended in 2 ml of low-salt buffer containing 20 mM HEPES pH 7.9, 10 mM KCl, 0.1 mM NaVO_4_, 1 mM EDTA, 1 mM EGTA, 0.2% NP-40, 10% glycerol, complete protease inhibitor (Roche Applied Science), and incubated for 10 min on ice. Then the mixture was homogenized with glass Dounce homogenizer, centrifuged for 1 min at 12 000 rpm at 4°C. The remaining pellet was dissolved with 200 µl high-salt buffer containing 420 mM NaCl, 20 mM HEPES pH 7.9, 10 mM KCl, 0.1 mM NaVO_4_, 1 mM EDTA, 1mM EGTA, 20% glycerol, and complete protease inhibitors. Samples were kept on ice for 30 min, then centrifuged for 10 minutes at 12 000 rpm at 4°C. Supernatants were taken as nuclear fractions. Protein concentration was measured with a BCA assay kit (Pierce, Rockford, IL) according to the manufacturer's instructions.

Antibodies against GR, Sp1 and p-CREB used for Western blot analysis were the same as used for ChIP assay. Images were captured using VersaDoc 4000MP system (Bio-Rad), and the band densities were calculated using Quantity-One software (Bio-Rad). Histone H1 (BS1656, Bioworld Technology, MN, USA) was used as a loading control. The nuclear contents of the transcription factors are presented as the fold change relative to LW.

### Statistical analysis

All data are presented as mean ± SEM (n = 6), and were analyzed using Independent-Samples T Test with SPSS 13.0 for Windows (SPSS, Inc., Chicago, IL). The mRNA abundances of the alternative first exons are expressed as the fold change relative to that of the exon 1–4 in LW piglets. Nuclear protein abundances are expressed as the fold-change relative to that in LW. * and ** indicate significant difference between breeds at *P*<0.05 and *P*<0.01, respectively.

## Supporting Information

Table S1 **Raw data from MassArray analysis of CpG islands of promoter of GR.**
(XLS)
